# The ins and outs of spinal cord stimulation

**DOI:** 10.1093/braincomms/fcae416

**Published:** 2025-01-15

**Authors:** Jason B Carmel

**Affiliations:** Department of Neurology, Columbia University, College of Physicians and Surgeons, New York, NY 10032, USA

## Abstract

This scientific commentary refers to ‘Intraspinal microstimulation of the ventral horn has therapeutically relevant cross-modal effects on nociception’, by Bandres *et al*. (https://doi.org/10.1093/braincomms/fcae280).

**This scientific commentary refers to ‘Intraspinal microstimulation of the ventral horn has therapeutically relevant cross-modal effects on nociception’, by Bandres *et al*. (https://doi.org/10.1093/braincomms/fcae280).**

Spinal cord stimulation has been FDA approved to treat chronic pain since the 1980s, and about 50 000 people receive these implants each year. More recently, spinal cord stimulation has emerged as a technique to improve movement in people with paralysis, including spinal cord injury,^1^ stroke^2^ and cerebral palsy.^3^ Epidural spinal cord electrodes are positioned over the dorsal spinal cord, and stimulation activates large-diameter afferents as they enter the spinal cord and in the dorsal columns.^4^ The treatment of both pain and paralysis with these dorsal electrodes begs the question about the role of ventral circuits in spinal cord stimulation.

In a recent issue of *Brain Communications*, Bandres *et al*.^5^ addressed the question of whether spinal cord stimulation in the ventral horn could reduce neuropathic pain transmission in a rat model of chronic spinal cord injury. First, rats were subjected to thoracic spinal cord injury, which causes neuropathic pain. In a terminal physiology experiment, a sharp multielectrode was inserted into the lumbar spinal cord. An electrode near the multielectrode tip was positioned in the ventral horn to deliver intraspinal microstimulation for neuromodulation. More proximal electrodes were used to record neurons in the dorsal horn while providing mechanical and nociceptive stimulation to the hindlimb. The authors found that microstimulation was effective in reducing nociceptive sensory feedback in a subgroup analyses of nociceptive specific and wide dynamic range neurons, primarily via a preferential modulation of nociceptive transmission. Further analyses suggested that this modulation is unlikely due to changes in spinal excitability in the dorsal horn. Non-nociceptive transmission was unaffected. The findings are similar to a previous publication in which the authors performed intraspinal stimulation on naïve rats.^6^ This study represents a next logical step to understand how spinal stimulation affects sensory transmission in rats with the neuropathic condition of spinal cord injury.

A critical question left by this study and others using intraspinal stimulation is the specificity of the stimulation to the stimulated circuits. Focal stimulation suggests focal activation, but interconnected circuits are also activated. The connections between the largely motor ventral circuits and the largely sensory dorsal circuits are famously robust, serving as a test case for the elucidation of circuits through reflex testing.^7^ Here, a comparison of ventral stimulation to the more common dorsal stimulation could be revealing. Perhaps the comparison would show specific ventral effects. Alternatively, the effects of ventral versus dorsal stimulation could be more similar than their classical parcellation into motor versus sensory functions would predict. This seems to be the case for dorsal stimulation, which can reduce pain and improve movement.

There are other important steps for the ventral stimulation to be proven as the multi-modal therapy that the authors envision. First, all the experiments in the current study were conducted in anaesthetized rats. Creating methods for ventral stimulation in awake animals will be necessary to determine whether such stimulation alters neuropathic pain behaviours. In addition, while ventral stimulation might help with movement recovery, this has not been tested, and the studies demonstrating functional recovery in humans have been with dorsal stimulation. The predominant use of dorsal stimulation is likely due to the dorsal epidural space being more surgically accessible. As ventral stimulating electrodes and their insertion techniques become easier,^8,9^ the utility of this approach and its relative efficacy compared with dorsal stimulation can be tested.

As systems neuroscientists and the neuromodulation community try to better understand the mechanisms of electrical spinal cord stimulation, the methods used by the McPherson group hold promise. One can envision the use of multielectrode recordings to measure the effects of various stimulation sites, including intraspinal, epidural or even transcutaneous.^10^ Separating the response of specific neurons from the large stimulation artefact is not trivial, but the input–output relationships are an important way to determine the circuits activated by stimulation. Other approaches will be to gain control over certain spinal populations, for example with optogenetic stimulation,^11^ or to manipulate the inputs to the spinal cord to understand that are necessary or sufficient for neuromodulation.^12^ As the evidence for spinal cord stimulation builds for recovery of movement, these mechanistic studies become more urgent. They may help to improve the location, pattern and intensity of stimulation for clinical efficacy.
